# Early Reassessment of Total Metabolic Tumor Volume on FDG-PET/CT in Advanced Melanoma Patients Treated with Pembrolizumab Predicts Long-Term Outcome

**DOI:** 10.3390/curroncol28030152

**Published:** 2021-04-27

**Authors:** Sim Vermeulen, Gil Awada, Marleen Keyaerts, Bart Neyns, Hendrik Everaert

**Affiliations:** 1 Department of Nuclear Medicine, Vrije Universiteit Brussel, Universitair Ziekenhuis Brussel, Laarbeeklaan 101, 1090 Jette, Belgium; marleen.keyaerts@uzbrussel.be (M.K.); hendrik.everaert@uzbrussel.be (H.E.); 2 Department of Medical Oncology, Vrije Universiteit Brussel, Universitair Ziekenhuis Brussel, Laarbeeklaan 101, 1090 Jette, Belgium; Bart.Neyns@uzbrussel.be

**Keywords:** Pembrolizumab, PET/CT, melanoma

## Abstract

PD-1 Immune checkpoint inhibitors, such as Pembrolizumab, can have a durable beneficial therapeutic effect in patients with advanced melanoma. However, not all patients will benefit equally from these therapies, and (potentially life-threatening) immune-related adverse events may occur. In this study, we investigate the value of early response assessment by FDG-PET/CT as a biomarker for predicting survival. We identified all patients with advanced melanoma who were treated with Pembrolizumab in our medical center and underwent a baseline and at least one follow-up FDG-PET/CT. The total metabolic tumor volume (TMTV) was calculated, and the evolution was compared to survival parameters. A total of 77 patients underwent a baseline and at least one follow-up FDG-PET/CT, 36 patients had follow-up imaging within 2–4 months, and 21 patients an FDG-PET/CT 5–6 months after baseline. When the TMTV evolution was categorized into two subgroups (stable/decrease versus increase), an association was found between stability or decrease in TMTV and better PFS and OS. A similar trend, however non-significant, was observed at 5–6 months. The evolution in TMTV as assessed by FDG-PET/CT 2–4 months after treatment initiation is associated with long-term outcomes in patients with advanced melanoma treated with Pembrolizumab.

## 1. Introduction

Malignant melanoma is a frequently occurring type of cancer and the incidence is increasing faster than that of other malignancies [1,2,3]. Immune checkpoint inhibitors (ICI’s) have been shown to be more effective in the treatment of advanced melanoma than conventional chemotherapy due to the high immunogenicity of the tumor [4,5,6,7]. Pembrolizumab is an example of an ICI in which the anti-tumoral immune response is triggered by blocking the interaction of programmed cell death 1 (PD-1) with its ligands PDL-1 and PDL-2.

The keynote 001, 002, and 006 studies show a response rate of 30–40% with Pembrolizumab monotherapy implying that not all patients will benefit from the therapy. Furthermore, patients treated with Pembrolizumab are at risk of developing immune-related adverse events. Early differentiation between responders and non-responders could help identify a subset of patients who will benefit from continuing treatment with Pembrolizumab, while sparing other patients from continuing futile and possibly toxic therapy. In line with the study by Tan et al. showing an association between metabolic remission on FDG-PET/CT (18-fluorodeoxyglucose positron emission tomography/computed tomography) one year after the start of anti-PD-1 therapy and outcome (response and survival), we investigated whether long-term outcome on Pembrolizumab monotherapy could be predicted based on metabolic tumor volume change on FDG-PET/CT implemented sooner after initiation of Pembrolizumab treatment [8]. The total metabolic tumor volume (TMTV) is a quantitative PET biomarker that reflects the metabolically active volume of a tumor and is semi-automatically calculated. A relationship has already been demonstrated between outcome and various clinical factors such as certain previous treatments (for example Ipilimumab and BRAF-/MEK- inhibitors), presence of brain metastases, and the number of metastatic locations. Immunohistochemical analysis also shows promising results for the prediction of outcomes [9].

The aim of this study is to investigate whether the evolution in total metabolic tumor volume assessed by early on-treatment FDG-PET/CT is associated with outcomes in patients with advanced melanoma treated with Pembrolizumab.

## 2. Materials and Methods

### 2.1. Study Design and Patient Population

Patients with stadium III and IV melanoma, according to the American Joint Committee on Cancer (AJCC), treated with Pembrolizumab monotherapy (2 mg/kg every 3 weeks) in the first- or later-line setting in the University Hospital of Brussels were included in this monocentric retrospective analysis. Only patients with cutaneous, mucosal melanoma, or melanoma with an unknown primary lesion were included; patients with uveal melanoma were excluded. The study population consisted of patients who received at least one administration of Pembrolizumab between 1 September 2014 and 3 September 2019. Follow-up data were collected until 29 March 2020. All patients underwent a baseline FDG-PET/CT before the first administration of Pembrolizumab. Only patients with a follow-up FDG-PET/CT 2–4 months after initiation of therapy and/or 5–6 months after initiation were included. Patients where treatment was discontinued due to immune-related adverse effects (irAE) or for other reasons between baseline FDG-PET/CT and follow-up FDG-PET/CT were excluded.

### 2.2. Acquisition and Image Analysis

For the imaging routine, FDG-PET/CT was used. Patients were instructed to fast at least 4 h prior to the examination and blood glucose levels had to be less than 200 mg/dL. In case of elevated blood glucose levels, short-acting insulins were administered and FDG injection was postponed until their effect had worn off (rising glycemic trend). The administered activity was based on body weight: >60 kg: 250 MBq, 60–80 kg: 275 MBq, >80 kg 300 MBq, and 200 MBq in bedridden patients. Imaging was performed 60 min after tracer injection. Full-body images (vertex-feet) were acquired.

Total metabolic tumor volumes (TMTV) were determined using Syngo.via software (Siemens Healthineers GmbH, Erlangen, Germany). The TMTV was calculated as the sum of all tumor-associated voxels with a standardized uptake value (SUV) above the mean SUV measured in a reference region in normal liver tissue plus three standard deviations (SD) (SUVmean liver + 3 SD) of tumor lesions sized ≥ 1 mL. Patients with a baseline TMTV of zero due to lesions not surpassing the set threshold were not taken included in the analysis.

### 2.3. Clinical Response Evaluation

Clinical responses were evaluated using the immune-related response criteria (irRC) [10]. Progressive free survival (PFS) was defined as the time between the date of FDG-PET/CT examination and progressive disease (PD) or death (whichever occurred first); Overall survival (OS) was defined as the time between FDG-PET/CT examination and death.

### 2.4. Statistical Analysis

In patients who had more than one follow-up FDG-PET/CT between 2–4 or 5–6 months after initiation of anti-PD-1-based therapy, the earliest follow-up FDG-PET/CT was selected, and other examinations were excluded from the database. The date of follow-up FDG-PET/CT was set as a landmark point for survival analysis, thus excluding patients who developed the progressive disease before or at the moment of the examination and therefore discontinued therapy.

Median PFS and OS (in weeks) were calculated using the Kaplan–Meier method. The log-rank (Mantel-Cox) test was used to compare survival between the subgroups. The cut-off *p*-value of significance was set at 0.05 for all analyses. IBM SPSS software was used (IBM, Armonk, NY, USA, version 27).

## 3. Results

### 3.1. Baseline Characteristics

We identified 183 patients with advanced melanoma who were treated with Pembrolizumab monotherapy of whom 112 patients had undergone baseline FDG-PET/CT; 77 patients had at least one follow-up imaging exam. At 2–4 months, 36 patients and at 5–6 months, 21 patients were included in our analysis. The baseline characteristics of these patient subgroups are summarized in Table 1.

### 3.2. Outcome

At the time of database lock (29 March 2020) 38 out of 77 patients had died (median OS 168.4 weeks (95% CI 62.0–275.0)). There was a median follow-up time of 117.9 weeks from baseline FDG-PET/CT (range 5.71–294.86) with an average of 11.1 administrations of Pembrolizumab (range 1–42). The median PFS was 28.4 weeks (95% CI 14.6–42.2). As of 29 March 2020, four patients were still receiving treatment with Pembrolizumab and in 73 patients, treatment was discontinued because of progressive disease (58.9%), irAE (12.3%), sustained complete response (CR) (19.2%), or by patients’ decision (9.6%).

### 3.3. Response Evaluation Based on PFS and OS Compared to TMTV Change

#### 3.3.1. PFS and OS Compared to TMTV Change between Baseline PET/CT and Follow-Up PET/CT after 2–4 Months

Total metabolic tumor volume on the first follow-up FDG-PET/CT after baseline examination was compared to TMTV on baseline PET/CT and the change in TMTV was subdivided into two categories: stable/decreased TMTV (26 patients, 72.2%) versus increased TMTV (10 patients, 27.8%).

**Figure 1:**
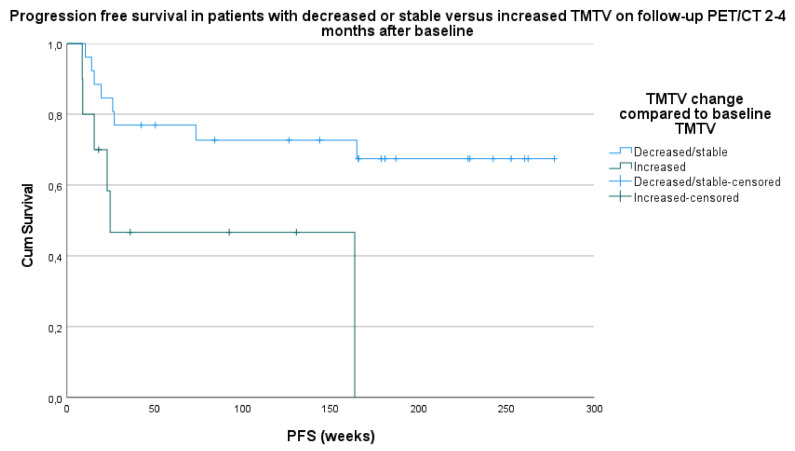
Survival analysis based on progressive free survival (PFS) in patients with stable/decreased TMTV versus increased TMTV on follow-up PET/CT 2–4 months after baseline compared to TMTV on baseline PET/CT.

Median PFS in the stable/decreased TMTV category was 203.1 weeks (95% CI 159.7–246.5). Median PFS in the increased TMTV category was 85.3 weeks (95% CI 33.0–137.5) (hazard ratio 5.5; *p* = 0.019) (Figure 1).

**Figure 2:**
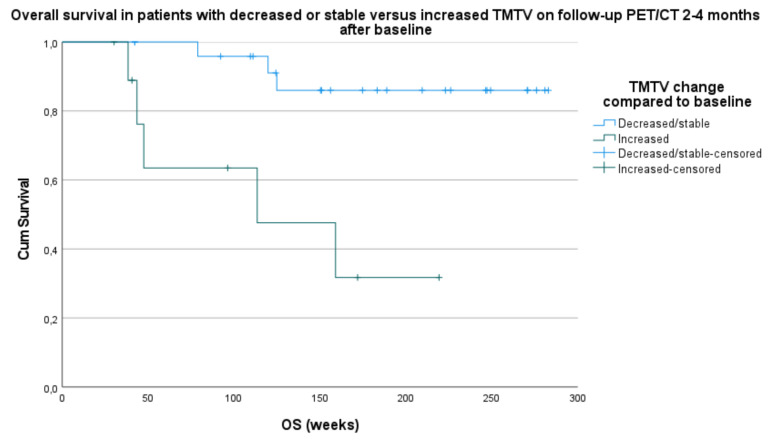
Survival analysis based on overall survival (OS) in patients with stable/decrease in TMTV versus increase in TMTV on follow-up PET/CT 2–4 months after baseline compared to TMTV on baseline PET/CT.

Median OS in the stable/decreased TMTV category was 258.6 weeks (95% CI 232.9–284.2) and 128.7 weeks in the increased TMTV category (95% CI 76.2–181.1) (HR 10.5; *p* = 0.001) (Figure 2).

**Figure 3:**
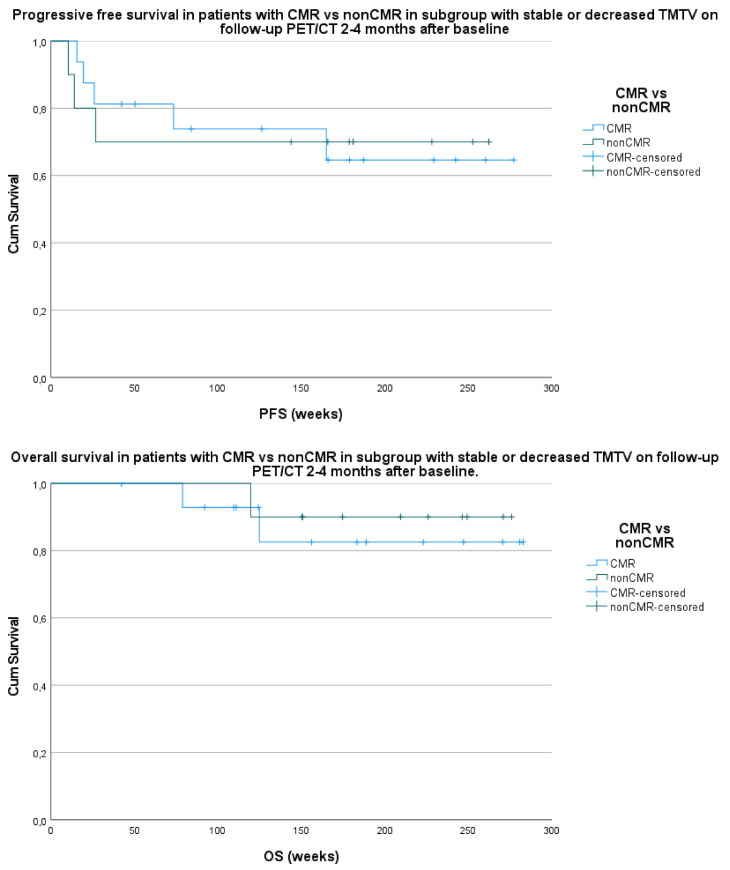
Comparison of survival parameters PFS (**above**) and OS (**below**) with patients with a complete metabolic remission (CMR) versus patients with residual disease or non-complete metabolic remission (nonCMR) and a stable or decreased total metabolic tumor volume (TMTV) on follow-up PET/CT 2–4 months after baseline PET/CT.

The patients with a stable or decreased TMTV in group 1 compared to baseline were further divided into patients with a complete metabolic remission (CMR) and patients with residual disease (or non-complete metabolic remission (nonCMR)) according to EORTC criteria [11]. Out of 26 patients, 16 (61.5%) with a decreased or stable TMTV in group 1 showed a CMR, and 10 out of 26 patients (38.5%) had residual disease. The comparison of these two groups with survival parameters PFS and OS failed to demonstrate a significant correlation with a p-value of respectively 0.993 and 0.629 (Figure 3).

There was no statistically significant correlation in the survival analysis with comparison of PFS and OS with TMTV when subdivided into six categories (stable TMTV, increase of 0–50%, 50–100%, and >100% in TMTV or decrease of 0–50% or 50–100% in TMTV). No significant results could be obtained from the comparison of PFS and OS with subdivision of TMTV changes in three categories (stable, increase, and decrease).

#### 3.3.2. PFS and OS Compared to TMTV Changes between Baseline PET/CT and Follow-Up PET/CT after 5–6 Months

A total of 21 patients had a follow-up FDG-PET/CT 5–6 months after baseline examination. Stable/decreased TMTV category consisted of 18 patients (85.7%) and increase in TMTV category of three patients (14.3%).

**Figure 4:**
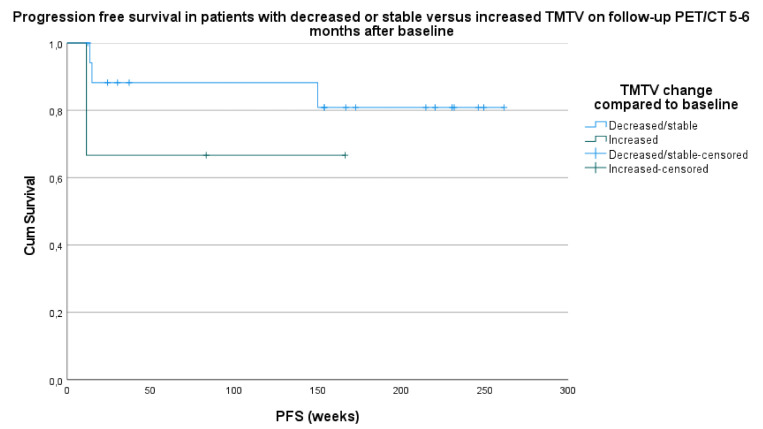
Survival analysis based on progressive free survival (PFS) in patients with stable/decrease in TMTV versus increase in TMTV on follow-up PET/CT 5–6 months after baseline compared to TMTV on baseline PET/CT.

Median PFS in the stable/decreased TMTV category was 224.3 weeks (95% CI 184.7–263.8) and 114.8 weeks (95% CI 32.2–197.4) in the increased TMTV category (HR 0.8; *p* = 0.364) (Figure 4).

**Figure 5:**
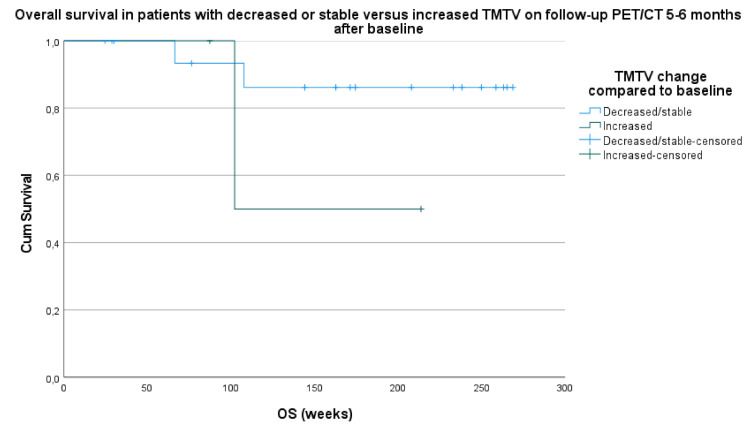
Survival analysis based on overall survival (OS) in patients with stable/decrease in TMTV versus increase in TMTV on follow-up PET/CT 5–6 months after baseline compared to TMTV on baseline PET/CT.

Median OS in the stable/decreased TMTV category was 243.7 weeks (95% CI 211.3–276.0) and 158.0 weeks (95% CI 80.6–235.2) in the increased TMTV category (HR 1.2; *p* = 0.267) (Figure 5). 

There was no statistically significant correlation in the survival analysis with the comparison of PFS and OS with TMTV when subdivided into six categories (stable TMTV, increase of 0–50%, 50–100%, and >100% in TMTV or decrease of 0–50% or 50–100% in TMTV). No significant results could be obtained from the comparison of PFS and OS with subdivision of TMTV changes into three categories (stable, increase, and decrease).

**Figure 6:**
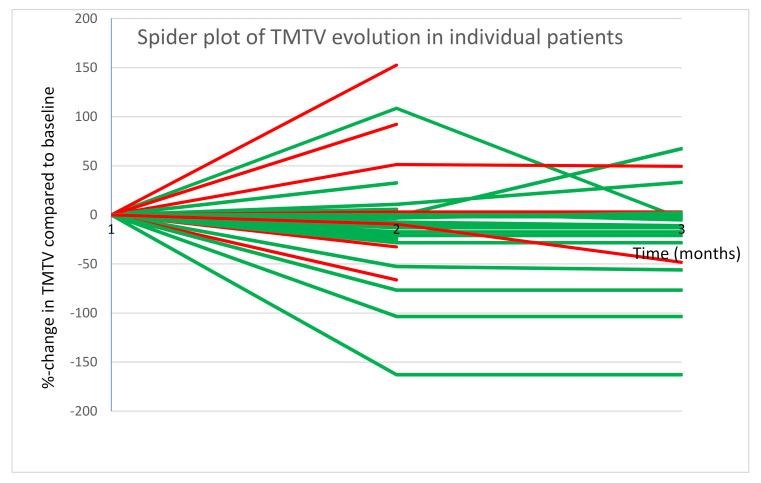
Spider plot demonstrating %-change in TMTV compared to baseline at 2–4 m and 5–6 m after onset of Pembrolizumab; Patients with PD within one year are shown in red and those without PD in green.

A spider plot was created demonstrating the change in TMTV (in %) compared to baseline at 2–4 months and 5–6 months after onset of Pembrolizumab. A color scale was used to make a distinction between patients who showed progressive disease within one year and those who remained progression free (Figure 6).

## 4. Discussion

The beneficial effect of immune checkpoint inhibitors on survival has already been demonstrated in the treatment of advanced malignant melanoma. Unfortunately, not every patient will respond to treatment. It is important to be able to distinguish early in the treatment course between patients who will respond and patients who will not benefit, not only because of the high costs and risk of immune-related side effects but also to spare them from ineffective therapy so that they can proceed to a next-line therapy. Many parameters have been examined in an attempt to demonstrate their relationship with treatment efficacy. It has already been shown that brain metastases have a negative impact on prognosis, especially when they are symptomatic and require treatment with corticosteroids for symptom control [12]. Also, high LDH levels, the number of metastatic sites, and previous treatment with systemic therapies have a negative effect. Other biochemical and immunohistochemical analyses are being studied to determine their capability to predict response to ICI and therefore outcome [9]. Our aim was to demonstrate whether changes in metabolic tumor volume assessed by FDG-PET/CT could be used to predict the long-term efficacy of Pembrolizumab.

The role of volumetric parameters in FDG-PET/CT as a predictive biomarker has been demonstrated in different types of cancers [5,13,14,15,16,17]. The total metabolic tumor volume is a valuable parameter as it reflects the extent and activity of the tumor [18,19,20]. In this study, we calculated the TMTV in follow-up FDG-PET/CT 2–4 and 5–6 months after baseline PET/CT in patients with an advanced melanoma treated with Pembrolizumab. We calculated the changes in TMTV between baseline and follow-up PET/CT and investigated the association with the therapeutic efficacy of ICI using survival analysis.

Patients who developed progressive disease per irRC before or at the moment of the follow-up examination and therefore discontinued therapy were excluded from this landmark analysis. However, some patients included in our analysis had an increase in TMTV and thus progressive disease according to the EORTC criteria, but did not interrupt therapy, as the treating physician could decide to discontinue ICI treatment only after confirmation of progressive disease, taking into account the possibility of pseudo-progression and delayed response that may occur with immunotherapy treatment. Of all patients with an increased TMTV in group 1 compared to baseline, treatment was continued due to favorable clinical response (5 out of 10), labeling them as having a stable disease by the treating physician (1 out of 10) or due to suspicion of pseudoprogression (4 out of 10). In the population of group 1 with an increase in TMTV (*n* = 10) compared to baseline, there were eventually five events (one progressive disease and four patients who died). In group 2, compared to baseline, there were three events, of which two were in the increase in TMTV subgroup and one in the stable or decreased TMTV subgroup, all due to death from melanoma progression. We could not demonstrate any predictive factors for these events. In four patients, treatment with Pembrolizumab, and thus follow-ups, were discontinued due to patients’ decision caused by a persistent metabolic response (50.0%) or due to irAE (50.0%).

We were able to demonstrate a significant association between stability or decrease in TMTV versus increase in TMTV between follow-up FDG-PET/CT 2–4 months after baseline and both PFS and OS.

The study of Tan et al. showed a response prediction based on TMTV one year after initiation of PD-1 immune checkpoint inhibition. Our results show that long-term response can already be predicted by analyzing the first follow-up FDG-PET/CT 2–4 months after the start of Pembrolizumab based on TMTV change. The same analysis for follow-up FDG-PET/CT after 5–6 months could not show any significant results, presumably due to a small sample size, but a similar trend was observed.

However, given the relatively small population, confirmation of our results is needed in larger populations. The retrospective aspect of the study and the fact that the results of FDG-PET/CT examination affected the clinician’s decision whether or not to continue therapy leads to a significant bias. It should also be noted that other landmark analyses (such as laboratory parameters, the evolution of brain metastases, tumor stage, amongst others) were not included in this study. A large prospective study with multivariable analysis could confirm our results leading to the possibility of fast recognition of non-responders and adaptation of the therapeutic strategy. It could, for example, be considered to associate CTLA-4 inhibitors to PD-1 ICI in patients with an increase in TMTV at first follow-up PET/CT to improve their outcome. This was an explorative analysis and confirmation of our findings is necessary using a large prospective study.

## 5. Conclusions

In our study, we found that TMTV change on follow-up FDG-PET/CT 2–4 months after baseline provides useful information to the clinician on PFS and OS which could support the decision whether or not to continue treatment with Pembrolizumab in patients with advanced melanoma. Patients with an increased total metabolic tumor volume on the first follow-up FDG-PET/CT were more likely to have a poor outcome. Based on these findings it could be considered to adapt the therapy (for example, associating CTLA-4 to PD-1 immune checkpoint inhibitors) to try to improve their outcome. Confirmation of our data in a large prospective analysis is needed.

## Figures and Tables

**Table 1:** Summary of baseline patient characteristics in all subgroups. Tumor stage was determined by the American Joint Committee on Cancer TNM 8th edition. Active brain metastases are defined as symptomatic.

Baseline Patient Characteristics	Patients with Baseline FDG-PET/CT	Patients with Follow-Up FDG-PET/CT 2-4 Months after Baseline	Patients with Follow-Up FDG-PET/CT 5-6 Months after Baseline
	*n* = 77	*n* = 36	*n* = 21
Subpopulation	Baseline	Group 1	Group 2
**Age**			
Median	66	65	62
Range	31–98	31–89	31–82
**Sex**			
Male	43 (55.8%)	18 (50.0%)	11 (52.4%)
Female	34 (44.2%)	18 (50.0%)	10 (47.6%)
**Melanoma subtype**			
Cutaneous	69 (89.6%)	33 (91.7%)	20 (95.2%)
Mucosal	3 (3.9%)	0 (0%)	0 (0%)
Unknown primary	5 (6.5%)	3 (8.3%)	1 (4.8%)
**Baseline WHO PS**			
0	49 (63.6%)	29 (80.6%)	15 (71.4%)
1	19 (24.7%)	5 (13.9%)	4 (19.1%)
2	9 (11.7%)	2 (5.6%)	2 (9.5%)
**Tumor Stage**			
IIIB	1 (1.3%)	1 (2.8%)	0 (0%)
IIIC	8 (10.4%)	7 (19.4%)	4 (19.1%)
IV-M1a	5 (6.5%)	3 (8.3%)	2 (9.5%)
IV-M1b	12 (15.6%)	6 (16.7%)	2 (9.5%)
IV-M1c	38 (49.4%)	16 (44.4%)	11 (52.4%)
IV-M1d	13 (16.9%)	3 (8.3%)	2 (9.5%)
**Brain metastases**			
Active	6 (7.8%)	0 (0%)	0 (0%)
Inactive	7 (9.1%)	3 (8.3%)	2 (9.5%)
**Number of affected organs**			
1			
2	21 (27.3%)	15 (41.7%)	8 (38.1%)
3	19 (24.7%)	10 (27.8%)	6 (28.6%)
>3	16 (20.8%)	6 (16.7%)	5 (23.8%)
	21 (27.3%)	5 (13.9%)	2 (9.5%)
**Number of prior therapies**			
0			
1	24 (31.2%)	14 (38.9%)	8 (38.1%)
2	30 (39.0%)	12 (33.3%)	5 (23.8%)
3	13 (16.9%)	6 (16.7%)	4 (19.1%)
>3	4 (5.2%)	1 (2.8%)	1 (4.8%)
	6 (7.8%)	3 (8.3%)	3 (14.3%)
**LDH level**			
<ULN	54 (70.1%)	27 (75.0%)	17 (81.0%)
≥ULN	23 (29.9%)	9 (25.0%)	4 (19.1%)
**BRAF^V600^ status**			
Mutant	34 (44.2%)	12 (33.3%)	8 (38.1%)
Wild type	43 (55.8%)	24 (66.7%)	13 (61.9%)
**TMTV**			
0	0 (0.0%)	16 (44.4%)	15 (71.4%)
>0–50	52 (67.5%)	15 (41.7%)	4 (19.1%)
(median, range)	(15.8, 1.0–49.5)	(10.7, 0.8–49.9)	(6.8, 3.8–10.8)
>50–100	9 (11.7%)	2 (5.6%)	1 (4.8%)
(median, range)	(66.2, 56.0–85.9)	(54.2, 50.5–58.0)	(52.0)
≥100	16 (20.8%)	3 (8.3%)	1 (4.8%)
(median, range)	(283.5, 103.5–1341.5)	(201.8, 116.4–336.4)	(126.7)

Abbreviations: WHO PS: World Health Organization Performance Status, LDH: lactate dehydrogenase, ULN: upper limit of normal, TMTV: total metabolic tumor volume.

## Data Availability

The data presented in this study are available on reasonable request from the corresponding author. The data are not publicly available due to ethical/privacy reasons.
